# Effect of the number of removed lymph nodes on prostate cancer recurrence and survival: evidence from an observational study

**DOI:** 10.1186/s12859-018-2180-8

**Published:** 2018-07-09

**Authors:** Chiara Gigliarano, Alessandro Nonis, Alberto Briganti, Marco Bonetti, Clelia Di Serio

**Affiliations:** 1 University of Insubria, Varese, Italy; 2 CUSSB, Vita-Salute San Raffaele University, Milan, Italy; 3 Ospedale San Raffaele, Milan, Italy; 4 Carlo F. Dondena Research Centre, Bocconi University, Milan, Italy

**Keywords:** Prostate cancer, Retrospective study, Doubly-robust estimation, Survival analysis

## Abstract

**Background:**

The aim of this article is to analyze the effect on biochemical recurrence and on overall survival of removing an extensive number of pelvic lymph nodes during prostate cancer surgery. The lack of evidence from randomized clinical trials to address this specific question has hampered the ability to determine the true effect of the number of nodes removed.

**Results:**

Our analysis is based on a large observational study, and this can lead unadjusted estimates to be very sensitive to confounding bias due to the different prognosis of individuals. We assess the effect of the number of lymph nodes removed by means of an Inverse Probability Weighting adjustment based on a Poisson regression model, and by a Doubly-robust adjustment.

**Conclusions:**

Our findings suggest that a large number of nodes removed is associated with a significant improvement in time to biochemical recurrence. However, it appears to have no impact on overall survival.

## Background

In prostate cancer (PCa) studies it is still debatable whether more extensive pelvic lymph node dissections are associated with better oncological outcomes. Nowadays, no prospective randomized study aimed at assessing the benefit on cancer control of anatomically defined extended pelvic lymph node dissection (PLND) as compared to limited or no PLND is indeed currently available.

Recent literature [1] reports that extended PLND is associated with higher rates of node positive findings, as the probability of detecting lymph node invaded by prostate cancer invasion is directly related to the extent of PLND. Another retrospective trial found a statistically significant association between the extent of PLND and cancer specific survival. The hazard of cancer-related death was found to be significantly lower for higher numbers of nodes removed [2]. The main limitation of this multicentre study is the lack of a homogeneous and standardized pathologic assessment of the removed lymph nodes. Given the long natural history of treated PCa, it is likely that if a potential beneficial effect of extended PLND existed, this is more likely to be detected in men with more aggressive cancer characteristics. Such hypothesis has been tested in more recent retrospective studies focusing on men with adverse pathological features. For example, Moschini et al. analyzed a large cohort of 1586 patients with locally advanced PCa treated with RP plus ePLND and found that a higher number of removed LNs was an independent predictor of increased cancer-specific survival, suggesting a potential therapeutic role of a more extensive PLNDs in this subgroup of patients [3].

Taken together, all of these data show that the impact of PLND as a curative treatment remains an open question. After the withdrawal of the only available prospective study reported on this topic [4], the scientific community is eagerly awaiting the results from the two ongoing randomized clinical trials comparing extended and limited PLND during RP in intermediate and high-risk PCa to provide definitive evidence to this highly controversial topic.

Overall, a general agreement exists that whenever PLND is indicated, this should be anatomically extended at least for diagnostic purposes [5, 6]. Nevertheless, whether this approach improves patient outcomes is still unknown. In case of detection of positive nodes at PLND, patients may be offered different post-operative adjuvant treatments, such as: (i) radiotherapy, (ii) hormone therapy, (iii) a combination of both. Correct patient staging may help in tailoring the optimal post-operative patient management and thus indirectly improve patient outcomes. Therefore, the correct identification of men potentially harboring prostate cancer nodal metastases is crucial to optimize patient outcome. Moreover, assessing whether such extensive dissections are directly associated with improved cancer control rates using methodologically sound approaches, which can account for the effect of patient selection biases, is still an unmet need.

## Methods

The aim of this article is to examine the effect on BCR and on all-causes t-month survival of removing different numbers of nodes during prostate cancer surgery. Our analysis is based on non-randomized observational study including 3046 PCa patients. In randomized studies the distributions of the patients characteristics are balanced across groups, so that groups are similar expect for the treatment. This constitutes a critical issue, since in non-randomized (or observational) studies treatment exposure may be associated with covariates that are also associated with the survival function, and thus “treated” patients may differ from “untreated” ones. We specify that in the paper we refer to “treatment” meaning the removal of a given number of lymph nodes. In this framework the usual analysis based on unweighted Kaplan-Meier survival curves may be misleading due to confounding [7].

Interpreting differences in groups or effects of covariates in observational studies is a major debatable point, since it is tempting to attribute the differences to the lack of random treatment assignment. Indeed, the lack of randomization can lead observational effect estimates to be very sensitive to confounding bias due to the different prognosis of individuals between treatment groups. As a consequence, observational randomized discrepancies cannot be automatically attributed to randomization itself.

Thus, observational studies need adjustments for baseline confounders, for time varying confounding an for selection bias. Guidelines that rely on observational data due to the absence of randomized trials benefit when the analysis mimics the analysis of a hypothetical target trial. To this extent inverse probability weight (IPW) can be considered as an adjustment for pre- or post-randomization variables in observational studies. In particular, in this article we use IPW to emulate the random allocation of patients to treatment groups (defined by the number of nodes removed), within a prostate cancer observational cohort study. We consider only pre-treatment variables for the adjustment.

The IPW approach, although being one of the most popular methods used for adjusting for confounding, is subject to some criticism. In particular, it tends to be sensitive to misspecification of the treatment assignment model. To overcome this problem we also exploit the Doubly robust method proposed by [8, 9], which tends to be more robust against misspecification either of the treatment assignment model or of the survival model. In the Appendix we report on a simulation study aimed at illustrating and exploring such properties for large and moderate sample sizes.

The rest of this paper is structured as follows. We first describe the data, and show by means of some preliminary analysis how the number of nodes removed clearly affects the probability of missing a positive-node patient modelled as beta binomial distribution. We then analyze whether the removal of more lymph nodes during prostatectomy may improve patient time from surgery to BCR and OS through better treatment assignment due to a more precise nodal staging assessment. For that reason, our analysis is split into two parts: the first part of our “Results” section focuses on time to BCR, while the second part reports the results of the analyses on overall survival (OS). Concluding remarks are reported in the “Discussion and conclusion” section.

## Results

### Data and patient classification

Our analysis is based on a prostate cancer dataset collected at San Raffaele Hospital (HSR) in Milan, Italy, between years 1991 and 2012. We consider a sample of 3046 patients with localized prostate cancer treated with radical prostatectomy and pelvic lymph node dissection.

The median follow-up is 38 months. During the entire follow-up period, 359 patients (12%) experienced BCR, while 84 patients (3%) died. Patients are aggregated in three groups according to the number of nodes removed during surgery: (i) 1-10 nodes, (ii) 11-20 nodes and (iii) more than 20 nodes removed. Preliminary descriptive statistics are shown in Table 1.

**Table 1:** Descriptive statistics

	Nodes group 1-10			Nodes group 11-20			Nodes group 21+			Total		
	Mean	Med	Freq	Mean	Med	Freq	Mean	Med	Freq	Mean	Med	Freq
Nodes removed	7.1	7		15.3	15		27.8	26		16.6	15.0	
Positive nodes	0.1	0		0.3	0		0.8	0		0.4	0.0	
Age (years)	64.8	65.4		65.4	65.9		64.7	64.8		65.0	65.0	
Gleason score	6.1	6		6.2	6		6.4	6		6.2	6.0	
PSA	8.8	6.9		11.6	7.1		12.3	7.1		11.1	7.0	
T1 stage			485 (*58%*)			756 (*55%*)			425 (*50%*)			1666 (*55%*)
T2 stage			281 (*34%*)			448 (*33%*)			288 (*26%*)			1017 (*33%*)
T3 stage			67 (*8%*)			163 (*11.9%*)			133 (*14.0%*)			363 (*11.9%*)
BCR (1 =yes)			93 (*11%*)			187 (*14%*)			79 (*9%*)			359 (*12%*)
OS (1 =dead)			19 (*2%*)			49 (*4%*)			16 (*2%*)			84 (*3%*)
N			833 (*27%*)			1367 (*45%*)			846 (*28%*)			3046 (*100%*)

Note: Med indicates the median of the variable; Freq refers to absolute frequencies (the corresponding percentage frequencies are in italics)

We note that almost half (45%) of the patients in the study belong to the second nodes group, with number of removed nodes between 11 and 20, and that the overall average number of nodes removed is 16.6. Table 1 also reveals that patients with larger numbers of nodes removed are more likely to have higher T stage, to be slightly younger, to have higher PSA values as well as higher Gleason scores than patients with fewer nodes removed. Therefore the distribution of the covariates does not seem to be independent from the treatment (the number of nodes removed). Moreover, the average number of positive nodes for all patients is 0.4, a value that is doubled for the groups of patients with more than 20 nodes removed. Hence, there seems to exist a positive association between the number of nodes removed and the number of positive nodes detected: the higher the number of nodes removed the higher is the probability of detecting a positive node.

For each *i*-th node-positive patient with number of removed nodes *Nex*_*i*_, let *X*_*i*_ be the number of observed positive nodes among the given number *Nex*_*i*_ of removed nodes, and *p*_*i*_ be the unknown probability that an examined node is positive.

The idea is to estimate the probability of missing a positive node (thus classifying incorrectly a patient - false negative) for a node-positive patient, based on the total number of removed nodes (*Nex*) and the number of nodes found positive (*x*).

The model assumes that *X* follows, conditionally on *p*, a binomial distribution with *Nex* (assumed independent) trials and success probability *p*. To allow for individual heterogeneity, it also assumes that *p* comes from a beta distribution with parameters *α* and *β*. The marginal distribution of *X* will be, therefore, the Beta-binomial distribution with parameters *α*,*β*,*Nex*, with probability mass function

$$\begin{array}{@{}rcl@{}} \lefteqn{P(X=x | Nex, \alpha,\beta)=} \\ && \left(\begin{array}{c} Nex \\ x \end{array} \right) \frac{Be(x+\alpha, Nex+ \beta -x)}{Be(\alpha,\beta)}, \end{array} $$

with *x*=0,1,...,*Nex* and where B(·,·) is the Beta function. For each patient with *Nex* examined nodes it is then possible to easily estimate the probability of observing no positive nodes as

$$P(X=0| Nex, \alpha, \beta)=\frac{\mathrm{B}(\alpha, Nex+ \beta)}{\mathrm{B}(\alpha,\beta)}.$$

This model relies on several assumptions which, although strong, are needed to ensure mathematical tractability. Among others, we are here assuming that our data do not contain incorrectly staged positive nodes, and that, within the same patient, all nodes have the same probability of being positive. For an extended discussion of the method and the reliability of those assumptions see Gönen et al. (2009).

**Fig. 1:**
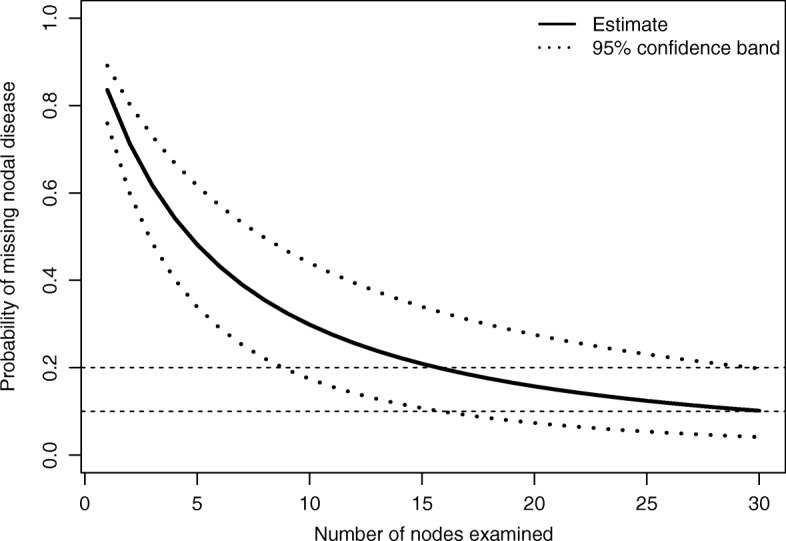
Estimated probability of missing nodal disease as a function of the number of nodes examined

We fitted a Beta-Binomial model to our data in R by maximization of the likelihood function, using the VGAM package v.1.0-4 [10]. The estimated parameters *α* and *β* led to the results showed in Fig. 1, together with a confidence band based on the endpoints of the marginal confidence intervals of the two parameters. The probability of not detecting the positive nodal status of a patient is plotted as a function of the number of nodes examined (*Nex*). The graph clearly shows that the probability of misclassifying node-positive patients decreases as the number of nodes removed increases. In particular, the probability of a false negative decreases quickly as *Nex* increases from 1 to 10, reaching a value around 0.3 for *Nex* =10. Starting from *Nex* =11, the curve becomes flatter. It reaches a value of 0.21 for *Nex* =15, and it remains between 0.2 and 0.1 with more than 15 removed nodes, with no further notable drop beyond that level. Considering also a clinical point of view, in terms of both feasibility of the procedure and quality of life of the patient that undergoes surgery, a removal of about 15 lymph nodes represents a good trade off to obtain a fair estimate about the real nodal status of a patient, while removing less than 10 lymph nodes may cause an excessively high false negative rate.

### Time to biochemical recurrence analysis

#### Unweighted survival analysis

We first perform an unweighted survival analysis, based on the Kaplan Meier estimation of the survival functions for each of the three node groups.

**Fig. 2:**
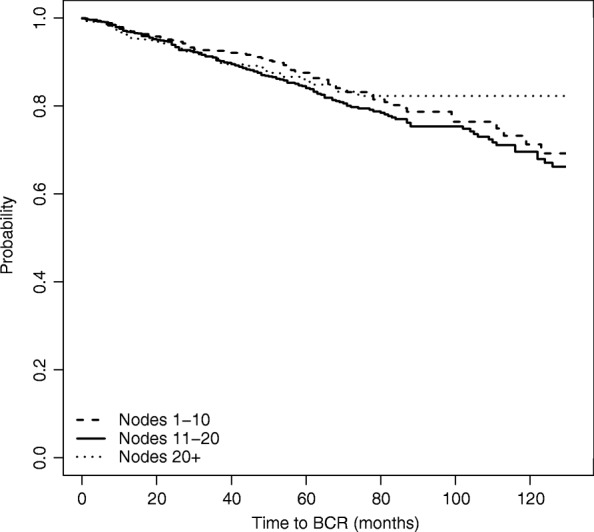
Estimated survival function for time to BCR, based on naive unadjusted estimation

Figure 2 plots the estimated survival curves for each group, while Table 2 shows the *p*-values of the log-rank tests for the difference in the survival functions between each pair of nodes groups. Both the graph and the tests indicate no differences among the three survival curves, thus suggesting that the number of nodes removed (*Nex*) does not seem to affect time to BCR.

**Table 2:** Log-rank tests for the difference in survival estimates among node groups - BCR

Node groups	Log rank test *p*-value
1-10 vs 11-20	0.116
1-10 vs 21+	0.718
11-20 vs 21+	0.265

However, since the study is observational as we have mentioned, the unweighted Kaplan-Meier survival curves may provide misleading results due to confounding: the apparent absence of differences across the groups might be in part explained by differences in the composition of the patient groups. If the experiment were randomized, then the number of nodes removed (that is, the treatment) would have been distributed in the same way across the patients, regardless of their covariates (PSA, Gleason score, age). In the previous section we have seen that this is actually not the case, since the surgeon’s decision was likely affected by these covariates. As more nodes are removed from patients with higher T stage, PSA and Gleason score, who also might be at higher risk of recurrence, the time to event distributions need to be adjusted for the effect of such confounding.

#### Inverse probability weighted analysis

A first possible method to adjust the analysis is based on the Inverse Probability Weights (IPW) adjustment, according to which each patient receives a weight that is inversely proportional to the estimated probability of having his number of nodes removed equal to the observed number; see, among others, [7, 11]. The IPW is a particular type of the propensity score method, which is aimed at adjusting for confounding by weighting observations by the inverse of the estimated propensity scores, that are probabilities of exposure to the treatment received conditionally on the covariates; see, among others, [12–15]. We have estimated the weights by fitting a Poisson regression model to estimate each patient’s probability of receiving any number of nodes removed. As covariates (*Z*) we have included age, PSA score, T-stage and Gleason score. We have also truncated the weights to a maximum of 35 (similarly to [16]).

More in detail, the IPW adjustment consists of the following steps:

- **Step 1.** Fit a Poisson regression model and compute the predicted probabilities of having the number of removed nodes be equal to the observed number given the covariates, *P*(*Nex*=*x*|*Z*_*i*_),*i*=1,…,*n*, so that *n* is the total number of patients. Then, sum over the three nodes groups to obtain the predicted probability of each node group *g*=1,2, or 3 and each covariate value:
  $$\begin{array}{@{}rcl@{}} P_{1}(Z_{i}) &=&\sum_{j=1}^{10} P(Nex = j | Z_{i}) \\ P_{2}(Z_{i})&=&\sum_{j=11}^{20} P(Nex = j | Z_{i}) \\ P_{3}(Z_{i})&=&\sum_{j=21}^{\infty} P(Nex = j | Z_{i}) \end{array} $$
- **Step 2.** Generate the Inverse Probability Weights (IPWs) for each patient as the inverse function of the corresponding predicted probability, which depends both on the number of nodes removed (*g*) and on the covariates:
  $$w_{g}(Z_{i})=\frac{1}{P_{g}(Z_{i})}, $$
  where *g* indicates the treatment group (1, 2, or 3).
- **Step 3.** Fit three weighted Kaplan Meier estimators with weights *w*_*g*_(*Z*_*i*_), separately for each nodes group: *S*_1_(*t*)=*P*(*T*≥*t*|*g*=1), *S*_2_(*t*)=*P*(*T*≥*t*|*g*=2) and *S*_3_(*t*)=*P*(*T*≥*t*|*g*=3).
- **Step 4.** Estimate the treatment effects in terms of difference in survival probabilities at time *t* among the three groups:
  $${S}_{1}(t) - {S}_{2}(t); {S}_{1}(t) - {S}_{3}(t); {S}_{2}(t) - {S}_{3}(t). $$
- **Step 5.** Estimate confidence intervals for the treatment differences using a bootstrap technique, for each time *t*.

The estimates from the Poisson regression are shown in Table 3, from which we notice that all covariates have a significant impact on the number of nodes removed.

**Table 3:** Output of Poisson regression

	Coefficient		Coefficient
Intercept	2.576 ^∗∗∗^	Gleason	0.045 ^∗∗∗^
T2 stage	0.038 ^∗∗∗^	PSA	0.002 ^∗∗∗^
T3 stage	0.147 ^∗∗∗^	Age	-0.003 ^∗∗∗^

^***^indicates statistical significance at the 0.01 level

**Fig. 3:**
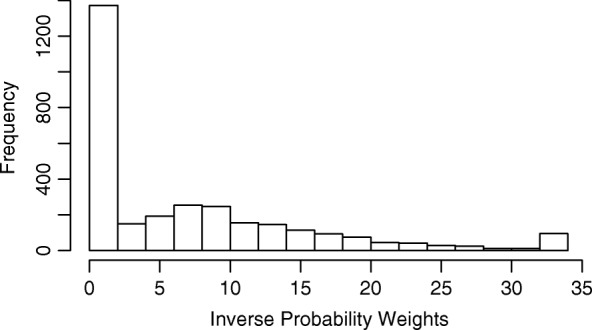
Histogram of the Inverse Probability Weights

**Fig. 4:**
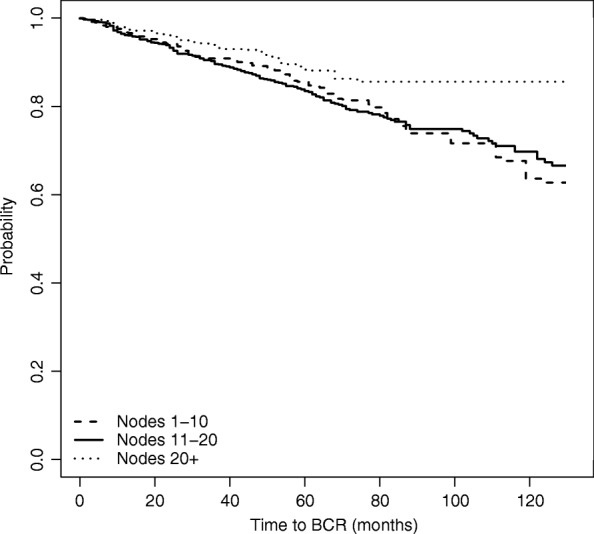
Estimated survival function for time to BCR, based on IPW estimation

Using the Inverse Probability Weights, whose distribution is shown in Fig. 3, we obtain the weighted Kaplan-Meier survival estimates shown in Fig. 4.

The graph reveals how the time to BCR curves would have looked like, had each of the node groups had the same covariates distribution. In particular, time to BCR seems to improve as the number of removed nodes increases.

Comparing Figs. 4 to 2 in the previous section we note that the estimated survival function of the group of patients with more than 20 nodes removed is higher in the IPW-adjusted Kaplan Meier than in the unweighted analysis. Moreover, no difference emerges between the survival distribution of patients with fewer than 10 nodes and the distribution for patients with 11-20 nodes removed.

Hence, once adjusting for confounding, we can conclude that there is a significant positive effect on time to BCR of removing a higher number of nodes.

#### Doubly robust analysis

An alternative way for adjusting for the non-randomized nature of treatment is provided by the Doubly robust method; see, among others, [8, 9, 17]. The IPW method tends to be possibly sensitive to misspecification of the treatment assignment model, while the Doubly robust method tends to be more robust against misspecification either of the treatment assignment model *P*(*Nex*=*x*|*Z*) or of the survival estimate model *S*(*t*|*Z*,*Nex*,*x*).

Indeed, while one will never know whether the weights are correctly specified, if that is the case then the survival probabilities are estimated consistently by IPW, as long as the model for the outcome is also correctly specified. The parameter estimators of the model for outcome, on the other hand, are not guaranteed to be consistent if that corresponding model is not correctly specified. So, IPW methods require that both models be correctly specified. Doubly robust methods, on the other hand, are theoretically guaranteed to consistently estimate the true survival probabilities even if one of the two models is wrongly specified (hence the name “robust”). In our setting, the survival estimates based on the Doubly robust method are consistent as long as either the Cox model or the weight (or both) models are correctly specified.

As stated in [9], “In a causal inference model, an estimator is Doubly Robust (DR) if it remains consistent when either (but not necessarily both) a model for treatment assignment mechanism or a model for the distribution of the counterfactual data is correctly specified.” “With observational data one can never be sure that a model for the treatment assignment mechanism or a model for the counterfactual data is correct.” “DR estimators, in contrast with inverse probability weighted-estimators, give the analyst two chances, instead of one, to make valid inference.” In that article, the authors also present the results of a simulation study that demonstrates the impressive finite sample performance of DR estimators in terms of finite sample efficiency and robustness. In [18], additional simulation based evidence is provided to quantify the theoretical properties of the Doubly Robust approach.

The Doubly robust method can be summarized in the following steps:

- **Steps 1. and 2.** Proceed analogously as in Step 1 and Step 2 of the IPW method.
- **Step 3.** Fit three weighted Cox models, with weights *w*_*g*_(*Z*_*i*_). In particular, we fit one model for each treatment group *g*=1,2,3, where group 1 refers to 1-10 nodes, group 2 refers to 11-20 nodes, and group 3 to 21+ nodes. For detailed notation see also Section 2.2 of Bang and Robins [9].
- **Step 4.** Compute three predicted survival functions at month *t* for every patient one for each treatment group: $\tilde {S}_{i}(t | Group=g, Z)$. In this way each patient is assigned three estimated survivals, one factual and the other two counterfactual.
- **Step 5.** For each group *g*, compute the average survival function at month *t* over all *n* patients:
  $$\tilde{S}_{g}(t)=\frac{1}{n}\sum_{i=1}^{n} \tilde{S}_{i}(t| Group={g}, Z). $$
- **Step 6.** Estimate the effects in terms of differences in survival at time *t* among groups:
  $$\tilde{S}_{1}(t) - \tilde{S}_{2}(t); \tilde{S}_{1}(t) - \tilde{S}_{3}(t);\tilde{S}_{2}(t) - \tilde{S}_{3}(t) $$
- **Step 7.** Estimate confidence intervals using bootstrap, for each time *t*.

**Fig. 5:**
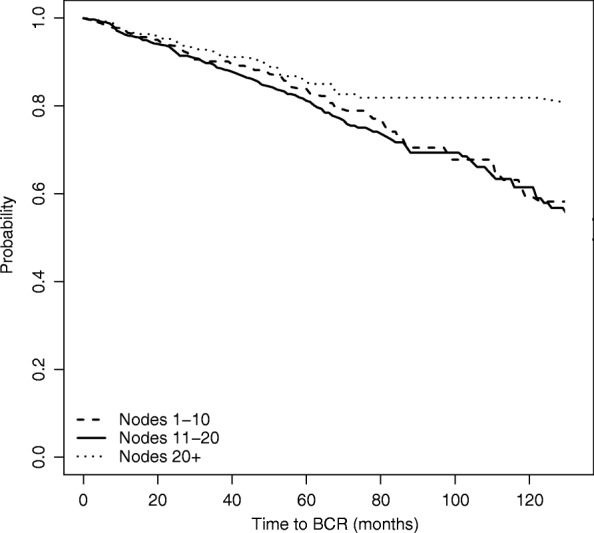
Estimated survival function for time to BCR, based on Doubly Robust estimation

Results are shown in Fig. 5. The graph confirms the results obtained with the IPW method, showing also in this case that the survival curve of the group of patients with highest number of removed nodes lies always above the survival curves of the other two groups, which are again identified as not being significantly different from each other.

Finally, in Table 4 we compare the three analyses that we have performed for estimating the group-specific time to BCR, focusing on *t*=60,90 and 120 months from surgery. The table shows that there is no difference across the three nodes groups in terms of probability of surviving more than 5 years (60 months) according to all estimation methods. This is also confirmed by the survival functions depicted in Figs. 2, 4 and 5. Differences between the high nodes group (21+ nodes) and the other groups start emerging from time *t*=90, that corresponds to the probability of surviving more than 7.5 years from the surgery. Consistently with earlier results, these differences emerge only when considering the two weighted models.

**Table 4:** Time to BCR

	Nodes group	t=60 months			t=90 months			t=120 months		
		Mean	95% C.I.		Mean	95% C.I.		Mean	95% C.I.	
Unweighted KM	1 (1-10)	0.866	0.836	0.898	0.764	0.710	0.822	0.692	0.612	0.782
	2 (11-20)	0.841	0.815	0.867	0.748	0.710	0.789	0.679	0.627	0.736
	3 (21+)	0.855	0.820	0.892	0.795	0.729	0.868	0.795	0.729	0.868
	1 vs 2	0.026	-0.015	0.066	0.016	-0.051	0.083	0.031	-0.048	0.111
	1 vs 3	0.011	-0.038	0.061	-0.031	-0.119	0.057	-0.047	-0.141	0.048
	2 vs 3	-0.014	-0.061	0.032	-0.047	-0.124	0.030	-0.078	-0.160	0.004
Weighted KM	1 (1-10)	0.847	0.839	0.855	0.717	0.701	0.732	0.627	0.605	0.650
	2 (11-20)	0.835	0.812	0.858	0.744	0.711	0.778	0.681	0.637	0.728
	3 (21+)	0.883	0.871	0.895	0.846	0.828	0.863	0.846	0.828	0.863
	1 vs 2	0.013	-0.032	0.058	-0.027	-0.102	0.047	-0.031	-0.119	0.057
	1 vs 3	-0.036	-0.092	0.020	-0.129	-0.232	-0.026	-0.161	-0.271	-0.050
	2 vs 3	-0.049	-0.104	0.006	-0.102	-0.197	-0.007	-0.130	-0.229	-0.031
Doubly Robust	1 (1-10)	0.839	0.791	0.887	0.705	0.605	0.805	0.592	0.432	0.752
	2 (11-20)	0.812	0.773	0.850	0.693	0.625	0.762	0.614	0.516	0.713
	3 (21+)	0.852	0.809	0.896	0.819	0.764	0.873	0.819	0.747	0.890
	1 vs 2	0.027	-0.035	0.089	0.012	-0.098	0.122	0.035	-0.112	0.182
	1 vs 3	-0.014	-0.080	0.052	-0.113	-0.222	-0.005	-0.141	-0.275	-0.007
	2 vs 3	-0.041	-0.103	0.021	-0.125	-0.225	-0.025	-0.176	-0.298	-0.054

The table contains, for each estimation method: (i) the estimated survival probabilities at 60, 90, and 120 months for each of the three treatment groups; (ii) the pairwise differences between the estimated survival probabilities across the three treatment groups. All estimated quantities come with bootstrap 95% confidence intervals

### Overall survival analysis

**Fig. 6:**
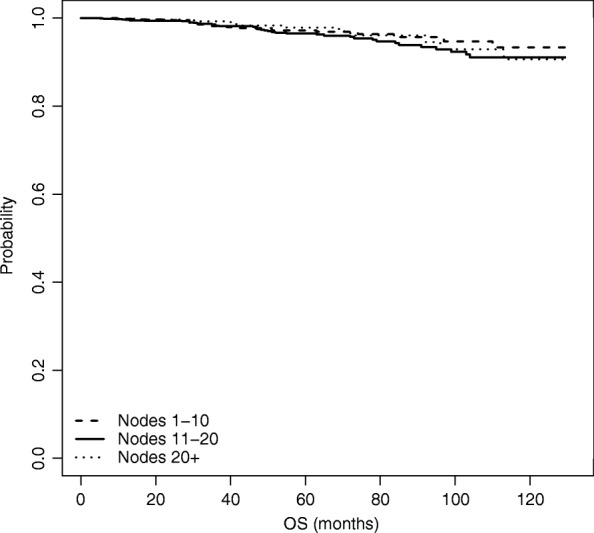
Estimated overall survival function, based on naive unadjusted estimation

We now move from time to BCR to time to OS, and repeat the same analyses as for time to BCR. Results of the naive unweighted analysis are shown in Fig. 6 and Table 5. Figure 6 plots the estimated survival curves for each group, while Table 5 shows the *p*-values of the unadjusted log-rank tests for the difference in the survival functions among each pair of nodes groups.

**Table 5:** Log-rank tests for the difference in survival estimates among node groups - OS

Node groups	Log rank test *p*-value
1-10 vs 11-20	0.13
1-10 vs 21+	0.797
11-20 vs 21+	0.255

Also for time to OS, both the graph and the tests show that there are no differences across the three survival curves, thus revealing that the number of nodes removed (*Nex*) does not seem to affect OS.

**Fig. 7:**
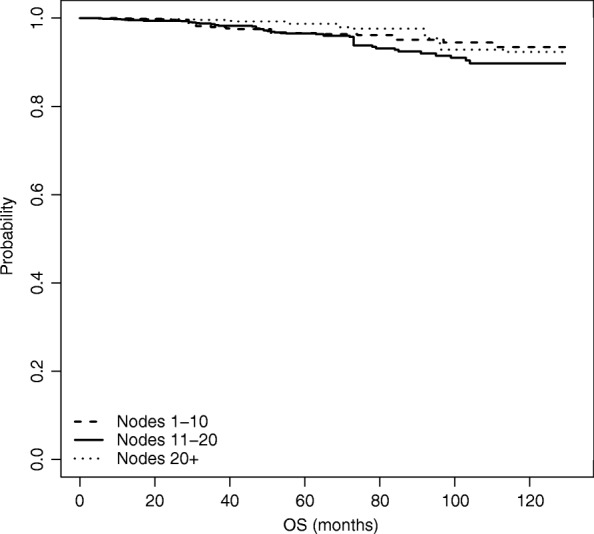
Estimated overall survival function, based on IPW estimation

**Fig. 8:**
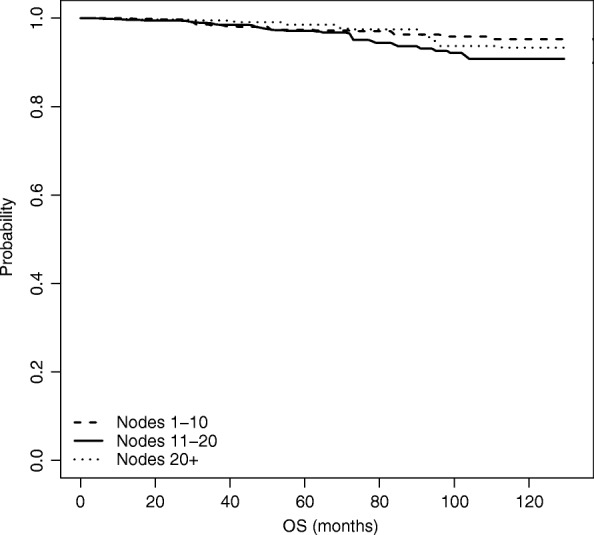
Estimated overall survival function, based on Doubly Robust estimation

We performed the weighted Kaplan-Meier estimation based on IPW as well as on the Doubly robust estimation; results are shown in Figs. 7 and 8, respectively. Differently from the analysis on time to BCR, in case of OS when we adjust for the presence of non-randomized study we do not see any significant differences; hence an increased number of nodes removed does not seem to significantly influence OS.

This is also confirmed by looking at Table 6, which compares the three analysis performed for the OS at *t*=60,90 and 110 months from surgery.

**Table 6:** Overall survival

	Nodes group	t=60 month			t=90 month			t=110 month		
		Mean	95% C.I.		Mean	95% C.I.		Mean	95% C.I.	
Unweighted KM	1 (1-10)	0.969	0.953	0.985	0.947	0.918	0.977	0.934	0.895	0.974
	2 (11-20)	0.963	0.948	0.977	0.934	0.910	0.959	0.900	0.863	0.939
	3 (21+)	0.970	0.947	0.993	0.945	0.905	0.987	0.906	0.842	0.975
	1 vs 2	0.006	-0.016	0.029	0.013	-0.025	0.051	0.033	-0.019	0.086
	1 vs 3	-0.001	-0.032	0.030	0.002	-0.049	0.053	0.027	-0.048	0.102
	2 vs 3	-0.007	-0.037	0.022	-0.011	-0.059	0.037	-0.006	-0.079	0.067
Weighted KM	1 (1-10)	0.963	0.959	0.968	0.945	0.938	0.952	0.935	0.926	0.944
	2 (11-20)	0.964	0.952	0.976	0.920	0.897	0.943	0.889	0.856	0.922
	3 (21+)	0.979	0.973	0.986	0.954	0.942	0.967	0.923	0.906	0.941
	1 vs 2	-0.001	-0.027	0.025	0.024	-0.019	0.067	0.046	-0.013	0.104
	1 vs 3	-0.016	-0.053	0.021	-0.009	-0.069	0.051	0.012	-0.073	0.096
	2 vs 3	-0.016	-0.050	0.019	-0.033	-0.090	0.023	-0.034	-0.118	0.050
Doubly Robust	1 (1-10)	0.974	0.956	0.991	0.963	0.930	0.996	0.952	0.896	1.008
	2 (11-20)	0.972	0.955	0.988	0.936	0.892	0.980	0.908	0.842	0.974
	3 (21+)	0.985	0.972	0.998	0.975	0.941	1.008	0.937	0.839	1.035
	1 vs 2	0.008	-0.069	0.085	0.038	-0.060	0.136	0.060	-0.068	0.188
	1 vs 3	-0.011	-0.090	0.067	-0.010	-0.121	0.100	0.020	-0.160	0.200
	2 vs 3	-0.020	-0.071	0.032	-0.048	-0.141	0.045	-0.040	-0.202	0.122

The table contains, for each estimation method: (i) the estimated survival probabilities at 60, 90, and 110 months for each of the three treatment groups; (ii) the pairwise differences between the estimated survival probabilities across the three treatment groups. All estimated quantities come with bootstrap 95% confidence intervals

After adjusting, no significant impact of the number of removed nodes on OS emerges for the three time points.

## Discussion and conclusion

Extended pelvic lymph node dissection (PLND) has certainly a key staging role in Prostate Cancer (PCa). Assessing the relative benefits and burden of PLND for oncological and non-oncological outcomes in patients undergoing radical prostatectomy for PCa is still debatable. The issue of whether PLND may affect prostate cancer outcomes such as progression and survival has been an argument of extreme interest in the urologic community over the last decades. Indeed, the impact of PLND on cancer outcomes remains controversial [1] due to the lack of prospective, randomized trials. The authors of [19] found a statistically significant negative association between the number of removed lymph nodes and BCR-free survival in patients with no positive nodes found. However, there is currently no available study supporting its role with regards to oncological outcomes in men with clinically localized disease. Thus, our approach represents a novel perspective to evaluate – within an observational setting – the effect on biochemical recurrence and on all-causes t-month survival of removing different numbers of nodes, providing important evidence also from non randomized trials.

We have shown how it is possible to assess the effect of the number of lymph nodes removed by means of an Inverse Probability Weighting adjustment (here, based on Poisson regression) and by a Doubly-robust adjustment method.

As a first analysis we had run traditional Cox models both for BCR and OS, with the number of nodes removed being classified in the three groups, and with the log-transformed original variable. The results of such standard analyses do not show an effect of the number of nodes removed (results not shown).

**Table 7:** Root Mean Squared Error (MSE) and Bias for the estimation of the marginal survival probability *S*(1000) for the three groups defined by the number of nodes received

Generation		Estimation					Unadjusted		Weighted		Doubly Robust	
PM	SM	PM	SM	n	Nodes	S(1000)	$\sqrt {\text {MSE}}$	Bias	$\sqrt {\text {MSE}}$	Bias	$\sqrt {\text {MSE}}$	Bias
1	1	1	1	1000	1-10	0.4808	0.0908	-0.0287	0.0987	-0.0060	0.0845	0.0093
					11-20	0.4808	0.0299	-0.0071	0.0292	-0.0004	0.0262	0.0011
					>20	0.4808	0.1113	0.0728	0.1002	0.0001	0.0935	0.0183
1	1	1	1	5000	1-10	0.4769	0.0459	-0.0259	0.0436	0.0002	0.0373	0.0027
					11-20	0.4769	0.0147	-0.0073	0.0128	-0.0005	0.0113	-0.0001
					>20	0.4769	0.0836	0.0756	0.0463	-0.0001	0.0417	0.0029
1	1	1	1	10000	1-10	0.4775	0.0356	-0.0235	0.0306	0.0005	0.0261	0.0017
					11-20	0.4775	0.0116	-0.0071	0.0093	-0.0002	0.0080	0.0000
					>20	0.4775	0.0786	0.0742	0.0336	-0.0007	0.0298	0.0013
[5pt] 2	2	2	2	1000	1-10	0.4108	0.0948	-0.0329	0.1067	-0.0060	0.0951	0.0102
					11-20	0.4108	0.0293	-0.0070	0.0288	-0.0005	0.0265	0.0005
					>20	0.4108	0.1091	0.0728	0.0968	0.0006	0.0927	0.0160
2	2	2	2	5000	1-10	0.4067	0.0498	-0.0312	0.0463	0.0003	0.0414	0.0031
					11-20	0.4067	0.0141	-0.0068	0.0125	-0.0002	0.0116	-0.0001
					>20	0.4067	0.0778	0.0697	0.0439	-0.0001	0.0410	0.0018
2	2	2	2	10000	1-10	0.4069	0.0391	-0.0289	0.0320	-0.0012	0.0292	0.0002
					11-20	0.4069	0.0111	-0.0064	0.0092	0.0002	0.0084	0.0002
					>20	0.4069	0.0739	0.0695	0.0322	0.0006	0.0300	0.0016
[5pt] 2	3	2	3	1000	1-10	0.4108	0.0948	-0.0329	0.1067	-0.0060	0.0951	0.0102
					11-20	0.2971	0.0283	-0.0044	0.0284	0.0001	0.0269	0.0014
					>20	0.5514	0.1056	0.0727	0.0978	0.0009	0.0895	0.0186
2	3	2	3	5000	1-10	0.4067	0.0498	-0.0312	0.0463	0.0003	0.0414	0.0031
					11-20	0.2941	0.0131	-0.0046	0.0125	-0.0000	0.0120	0.0001
					>20	0.5461	0.0764	0.0687	0.0443	0.0002	0.0390	0.0027
2	3	2	3	10000	1-10	0.4069	0.0391	-0.0289	0.0320	-0.0012	0.0292	0.0002
					11-20	0.2936	0.0099	-0.0045	0.0090	0.0000	0.0086	0.0001
					>20	0.5473	0.0708	0.0666	0.0317	-0.0001	0.0281	0.0015
[5pt] 3	2	3	2	1000	1-10	0.4108	0.1562	-0.1401	0.1061	-0.0082	0.0949	0.0100
					11-20	0.4108	0.0292	0.0011	0.0292	-0.0001	0.0264	0.0009
					>20	0.4108	0.1930	0.1710	0.1110	0.0099	0.0969	0.0241
3	2	3	2	5000	1-10	0.4067	0.1429	-0.1399	0.0455	-0.0023	0.0416	0.0008
					11-20	0.4067	0.0133	0.0010	0.0133	0.0000	0.0122	0.0002
					>20	0.4067	0.1754	0.1711	0.0490	0.0010	0.0414	0.0020
3	2	3	2	10000	1-10	0.4069	0.1362	-0.1345	0.0324	-0.0006	0.0300	0.0007
					11-20	0.4069	0.0095	0.0011	0.0094	0.0000	0.0086	0.0001
					>20	0.4069	0.1721	0.1698	0.0349	0.0002	0.0297	0.0008

Both models for propensity score and survival used in the estimation process here match the corresponding models used for data generation. Results are based on 3000 simulated samples. PM stands for Propensity Score Model, and SM stands for Survival Model. Please refer to the text for the description of the models

**Table 8:** Root Mean Squared Error (MSE) and Bias for the estimation of the marginal survival probability *S*(1000) for the three groups defined by the number of nodes received

Generation		Estimation					Unadjusted		Weighted		Doubly Robust	
PM	SM	PM	SM	n	Nodes	S(1000)	$\sqrt {\text {MSE}}$	Bias	$\sqrt {\text {MSE}}$	Bias	$\sqrt {\text {MSE}}$	Bias
2	2	3	2	1000	1-10	0.4108	0.0948	-0.0329	0.1144	0.0423	0.0929	0.0095
					11-20	0.4108	0.0293	-0.0070	0.0287	-0.0027	0.0265	0.0005
					>20	0.4108	0.1091	0.0728	0.1038	-0.0715	0.0895	0.0159
2	2	3	2	5000	1-10	0.4067	0.0498	-0.0312	0.0668	0.0483	0.0398	0.0029
					11-20	0.4067	0.0141	-0.0068	0.0126	-0.0023	0.0116	-0.0001
					>20	0.4067	0.0778	0.0697	0.0813	-0.0747	0.0377	0.0011
2	2	3	2	10000	1-10	0.4069	0.0394	-0.0294	0.0569	0.0471	0.0283	0.0000
					11-20	0.4069	0.0112	-0.0064	0.0094	-0.0021	0.0085	0.0002
					>20	0.4069	0.0735	0.0692	0.0809	-0.0776	0.0275	0.0006
[5pt] 2	3	3	3	1000	1-10	0.4108	0.0948	-0.0329	0.1144	0.0423	0.0929	0.0095
					11-20	0.2971	0.0283	-0.0044	0.0282	-0.0010	0.0269	0.0014
					>20	0.5514	0.1056	0.0727	0.1250	-0.0866	0.0882	0.0173
2	3	3	3	5000	1-10	0.4067	0.0507	-0.0325	0.0657	0.0468	0.0397	0.0020
					11-20	0.2941	0.0131	-0.0044	0.0125	-0.0008	0.0121	0.0003
					>20	0.5461	0.0769	0.0690	0.1004	-0.0923	0.0373	0.0022
2	3	3	3	10000	1-10	0.4069	0.0391	-0.0289	0.0575	0.0478	0.0279	0.0001
					11-20	0.2936	0.0099	-0.0045	0.0090	-0.0010	0.0086	0.0001
					>20	0.5473	0.0708	0.0666	0.1008	-0.0969	0.0270	0.0013
[5pt] 2	3	2	4	1000	1-10	0.4108	0.0948	-0.0329	0.1067	-0.0060	0.0920	0.0186
					11-20	0.2971	0.0283	-0.0044	0.0284	0.0001	0.0270	0.0082
					>20	0.5514	0.1056	0.0727	0.0978	0.0009	0.1013	-0.0114
2	3	2	4	5000	1-10	0.4067	0.0498	-0.0312	0.0463	0.0003	0.0413	0.0056
					11-20	0.2941	0.0131	-0.0046	0.0125	-0.0000	0.0134	0.0069
					3	0.5461	0.0764	0.0687	0.0443	0.0002	0.0470	-0.0029
2	3	2	4	10000	1-10	0.4069	0.0391	-0.0289	0.0320	-0.0012	0.0290	0.0019
					11-20	0.2936	0.0099	-0.0045	0.0090	0.0000	0.0105	0.0065
					>20	0.5473	0.0708	0.0666	0.0317	-0.0001	0.0335	-0.0024

Either the propensity score model or the survival model (but not both) used in the estimation process is different from the corresponding model used for data generation. Results are based on 3000 simulated samples. PM stands for Propensity Score Model, and SM stands for Survival Model. Please refer to the text for the description of the models

Our use of a Poisson regression model was motivated by the very nature of the treatment of interest here, clearly a counting variable. Using a multinomial distribution would have forced (at most ordinal) categories onto that variable. That would be a natural choice in presence of multiple (unordered) treatments, when a fully categorical analysis is appropriate (see, e.g., Feng et al., [20]). In our setting, such a classification would also have led to an increase in the number of parameters to be able to model all odds ratios with respect to the covariates potentially influencing the number of nodes extracted. Discretizing the problem to just a binary treatment outcome would have forced an even stronger classification of the treatment variable. So our choice was to allow for a parsimonious model for the number of nodes through the Poisson regression model, and to then consider (ordinal) groups for the estimation process. The reasons for such a two-step approach are the need to allow for easy interpretability and description of the results, while still keeping a moderate number of patients in each group for the estimation, while maintaining a modeling approach coherent with the nature of the underlying treatment variable. Here, “moderate number” is meant as a number of patients that allowed us to obtain informative 95% confidence intervals for the reported differences in survival probabilities across treatment groups. As a last comment, note that IPW could be very sensitive to the presence of extreme weights [16]. We truncated the weights to a maximum of 35, but we also performed the estimation with a truncation value of 20, with no apparent changes in the results.

Our findings suggest that a large number of nodes removed may be associated with a significant improvement in the time to BCR but there is no detectable impact on OS. However, the lack of any benefit of the extent of pelvic lymph node dissection on patient OS could also be related to the relatively short median follow-up of our population.

## Appendix

### Simulation study

In this Appendix we report on a simulation experiment designed to illustrate and explore the finite-sample and the large-sample properties of the estimation procedures that we have used.

We simulated data from the following setup. Covariates were generated for *n* *i*.*i*.*d*. subjects as

- $\sqrt {\text {PSA}} \sim N(2,2)$
- Age ∼*N*(60,9)
- Stage ∼ Multinomial(1,(.22,.33,.45)), i.e. taking the three values 1, 2, and 3
- (Number of nodes examined - 1) ∼ Poisson(*λ*_*PM*_(**z**_*i*_)). Treatment group (*Tx*) was then set equal to 1 (1-10 Nodes), 2 (11-20 Nodes) or 3 (>20 Nodes).

The vector **z**_*i*_ indicates the covariate vector for subject *i*, excluding the treatment group *Tx*_*i*_, and *λ*_*PM*_(**z**_*i*_) indicates the mean parameter defined as


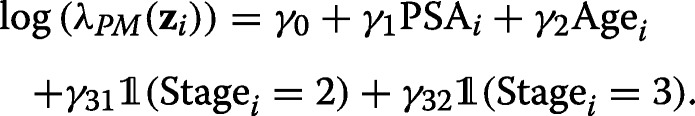


The propensity score models PM were defined as follows (in parentheses the values of the regression parameters):


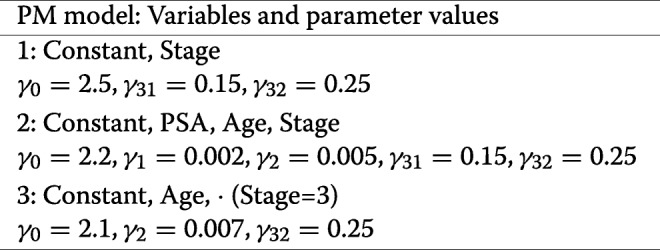


and covariates absent from a specific model were multiplied by a coefficient equal to zero in the generation step, and not estimated in the estimation process.

Conditionally on the covariates (including now the number of nodes examined, as classified in *Tx*), we generated survival times according to an exponential Cox proportional hazards model. The parameter *λ*_*SM*_(*t*|**z**_*i*_) of the exponential distribution for subject *i* was defined as


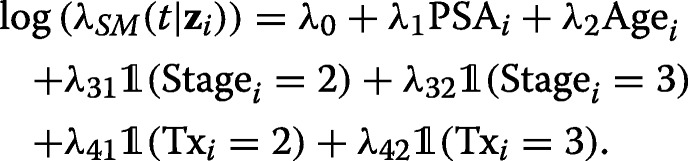


We considered the following four survival models SM (in parentheses the values of the regression parameters):


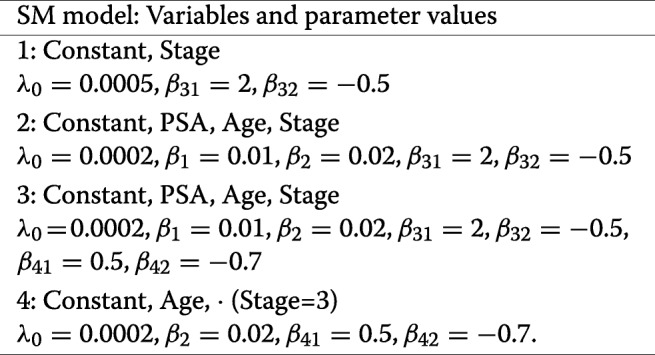


Here, too, covariates absent from a specific model were multiplied by a coefficient equal to zero in the generation step, and not estimated in the estimation process. The generated survival times were right censored with an independent censoring variable distributed as a negative exponential random variable with parameter 1/500.

**Table 9:** Root Mean Squared Error (MSE) and Bias for the estimation of the marginal survival probability *S*(1000) for the three groups defined by the number of nodes received

Generation		Estimation					Unadjusted		Weighted		Doubly Robust	
PM	SM	PM	SM	n	Nodes	S(1000)	$\sqrt {\text {MSE}}$	Bias	$\sqrt {\text {MSE}}$	Bias	$\sqrt {\text {MSE}}$	Bias
2	3	3	4	1000	1-10	0.4108	0.0948	-0.0329	0.1144	0.0423	0.1175	0.0671
					11-20	0.2971	0.0283	-0.0044	0.0282	-0.0010	0.0258	0.0051
					3	0.5514	0.1056	0.0727	0.1250	-0.0866	0.1015	-0.0641
2	3	3	4	5000	1-10	0.4067	0.0498	-0.0312	0.0668	0.0483	0.0733	0.0589
					11-20	0.2941	0.0131	-0.0046	0.0124	-0.0010	0.0120	0.0039
					3	0.5461	0.0764	0.0687	0.1005	-0.0926	0.0823	-0.0751
2	3	3	4	10000	1-10	0.4069	0.0391	-0.0289	0.0575	0.0478	0.0643	0.0565
					11-20	0.2936	0.0099	-0.0045	0.0090	-0.0010	0.0088	0.0034
					3	0.5473	0.0708	0.0666	0.1008	-0.0969	0.0840	-0.0804

Both the propensity score model and the survival model used in the estimation process differ from the corresponding models used for data generation. Results are based on 3000 simulated samples. PM stands for Propensity Score Model, and SM stands for Survival Model. Please refer to the text for the description of the models

**Table 10:** Estimated marginal survival probability *S*(1000) for thee three groups defined by the number of nodes received

Generation		Estimation				Unadjusted	Weighted	Doubly Robust
PM	SM	PM	SM	Nodes	S(1000)	Est Surv	Est Surv	Est Surv
1	1	1	1	1-10	0.4740	0.4522	0.4749	0.4751
				11-20	0.4740	0.4669	0.4739	0.4738
				>20	0.4740	0.5476	0.4743	0.4740
2	2	2	2	1-10	0.4037	0.3767	0.4042	0.4040
				11-20	0.4037	0.3974	0.4041	0.4041
				>20	0.4037	0.4734	0.4035	0.4035
2	3	2	3	1-10	0.4037	0.3767	0.4042	0.4040
				11-20	0.2911	0.2864	0.2910	0.2909
				>20	0.5438	0.6100	0.5427	0.5427
3	2	3	2	1-10	0.4037	0.2693	0.4014	0.4015
				11-20	0.4037	0.4047	0.4037	0.4037
				>20	0.4037	0.5756	0.4031	0.4032
2	2	3	2	1-10	0.4037	0.3767	0.4536	0.4040
				11-20	0.4037	0.3974	0.4017	0.4041
				>20	0.4037	0.4734	0.3261	0.4038
2	3	3	3	1-10	0.4037	0.3767	0.4536	0.4040
				11-20	0.2911	0.2864	0.2899	0.2909
				>20	0.5438	0.6100	0.4454	0.5430
2	3	2	4	1-10	0.4037	0.3767	0.4042	0.4046
				11-20	0.2911	0.2864	0.2910	0.2972
				>20	0.5438	0.6100	0.5427	0.5422
2	3	3	4	1-10	0.4037	0.3767	0.4536	0.4605
				11-20	0.2911	0.2864	0.2899	0.2941
				>20	0.5438	0.6100	0.4454	0.4613

Estimates are based on one sample of size 10 millions. PM stands for Propensity Score Model, and SM stands for Survival Model. Please refer to the text for the description of the models

Propensity scores for the three treatments (1-10, 11-20, and >20 Nodes examined) were computed as described in the main text, i.e. by adding the appropriate terms of the Poisson probability mass function. The theoretical survival probabilities were computed as the average over the observed covariate distribution (fixed for each simulation) of the quantities


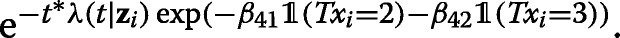


We estimated the survival probability at *t*^∗^=500,1000, and 1500 days. For simplicity, below we only report the results for *t*^∗^=1000 days. The conclusions drawn from for the other two time points were analogous.

Tables 7, 8 and 9 show the empirical root-MSE and Bias in estimating *S*(1000) with the naive Kaplan-Meier estimator (“Unadjusted”), with the weighted nonparametric estimator (“Weighted”), and with the Doubly robust estimator (“Doubly Robust”). Simulations were based on 3000 samples, and for sample sizes 1000, 3000, and 10000. Data were simulated and estimated by using different combinations of models PM and SM for the generation (“Generation”) and for the estimation (“Estimation”) processes.

Results show that the naive estimator is always biased. On the other hand, when both models used for estimation match the models used to estimate the data (i.e. they are the *correct* models), then both the weighted and the Doubly robust methods estimate the survival probabilities properly, with Bias and MSEs that tend to zero as the sample size increases. When only the propensity score is correctly specified, then both estimators are known to be consistent, and this is confirmed by the results. If, on the other hand, only the survival model is correctly specified, then the weighted estimator is clearly biased (as expected), while the Doubly robust estimator still shows its consistency. Lastly, when both models are wrongly specified, both estimators are clearly shown to be biased.

Interestingly, for the smaller sample size (1000) the MSE of the (biased) naive estimator is quite similar to that of the adjusted estimators, even when the latter two are known to be consistent. This implies that the former estimator has misleadingly smaller variance that the adjusted estimators. As sample size increases, as expected, the unadjusted estimator maintains a larger MSE than the other two estimators. Note that in the limit the MSE should become equal to the squared bias term (since the variance term tends to zero – recall that the MSE is equal to the sum of the variance of the estimator and the squared bias term). Indeed, for the n =10000 cases one can see that the absolute value of the Bias term of the unadjusted estimator approaches the value of the square root of the MSE.

For completeness, Table 10 shows the results of the three estimation procedures when they are applied, for each combination of propensity score and survival models, to just one sample of size n =10 million. That table again confirms the impressions gained from Tables 7, 8 and 9, and described above.

Note: Simulations were performed using the seed 13794 in R version 3.3.2.
